# *Vibrio harveyi* Infection Significantly Alters Amino Acid and Carbohydrate Metabolism in Whiteleg Shrimp, *Litopenaeus vannamei*

**DOI:** 10.3390/metabo10060265

**Published:** 2020-06-25

**Authors:** Seohee Ma, Ahran Kim, Wonho Lee, Seonghye Kim, Sujin Lee, Dahye Yoon, Jin-Sol Bae, Chan-Il Park, Suhkmann Kim

**Affiliations:** 1 Department of Chemistry, Center for Proteome Biophysics, and Chemistry Institute for Functional Materials, Pusan National University, Busan 46241, Korea; metabomsh@pusan.ac.kr (S.M.); ahran110@pusan.ac.kr (A.K.); wonholee@pusan.ac.kr (W.L.); seonghyeee@pusan.ac.kr (S.K.); isujin@pusan.ac.kr (S.L.); dahyeyoon@korea.kr (D.Y.); 2 Department of Herbal Crop Research, National Institute of Horticultural and Herbal Science, Rural development administration (RDA), Eumseong 27709, Korea; 3 Department of Marine Biology and Aquaculture, College of Marine Science, Gyeongsang National University, Tongyeong 53064, Korea; baejs0122@korea.kr (J.-S.B.); vinus0317@gmail.com (C.-I.P.); 4 National Fishery Products Quality Management Service (NFQS), 337, Haeyang-ro, Yeongdo-gu, Busan 49111, Korea

**Keywords:** high resolution-magic angle spinning, nuclear magnetic resonance spectroscopy, metabolomics, *Litopenaeus vannamei*, *Vibrio harveyi*

## Abstract

*Vibrio harveyi* is one of the pathogens that threaten the shrimp farming industry. However, metabolic changes induced by *V. harveyi infection* in shrimp remain unknown. In this study, we first conducted high resolution-magic angle spinning (HR-MAS) nuclear magnetic resonance (NMR)-based metabolomics studies on gill, hepatopancreas, and haemolymph of *V. harveyi*-infected white leg shrimp, *Litopenaeus vannamei*. Using multivariate statistical analysis, we observed a clear separation between the early (3 and 9 h post-injection (hpi)) and late phases (24, 72 and 144 hpi) of the infection in all tissues. Moreover, metabolic changes in response to *V. harveyi* infection were faster in the haemolymph in the early phase and significantly changed in the late phase of the infection in the gills. Extensive changes were observed in the hepatopancreas, with 24 hpi being the turning point of progression from early to late phase infection in the hepatopancreas. *V. harveyi infection* increased the energy demand in *L. vannamei* and the amino acid and carbohydrate metabolism pathways also exhibited significant changes depending on the tissue. Thus, each tissue displayed different metabolic changes, depending on the progress of the infection.

## 1. Introduction

Whiteleg shrimp (*Litopenaeus vannamei*) is the main marine species cultured in many developing countries, accounting for approximately 53% of the total crustacean production [1]. However, the shrimp industry has suffered from frequent outbreaks of epidemics caused by *Vibrio* spp. and various viruses [2]. The gram-negative bacteria of the genus *Vibrio* are ubiquitous in the aquatic environment [3] and *Vibrio harveyi* is an infectious agent that threatens the shrimp industry, having had a serious negative economic impact worldwide over the past 20 years [4,5]. Although *V. harveyi* causes diseases in a broad range of marine animals, such as abalone [6], sea bass [7], mussel [8], and shrimp [9,10], previous studies have focused on the histopathology, growth performance, and immune response of affected organisms. In addition, few studies have evaluated the metabolism of organisms infected with *V. harveyi* and, therefore, its effects on the metabolism remain unclear.

Metabolomics is an emerging field of ‘‘omics’’ studies that focuses on comprehensive and simultaneous profiling of the total metabolites in an organism [11]. Metabolites are the end products of cellular functions and are highly sensitive to environmental changes, such as disease or exposure to toxic compounds [12]. Nuclear magnetic resonance (NMR) spectroscopy and mass spectrometry have been the most frequently used analysis platforms in metabolomics studies. NMR enables the simultaneous detection of a wide range of structurally diverse metabolites, offering a metabolic “snapshot” at a given time-point [13]. NMR-based metabolomics has been used to study physiological variations in experimental animals and was proven to be a robust and reliable technique with high reproducibility in metabolomics studies of marine organisms [14,15,16,17,18]. Especially, high resolution-magic angle spinning (HR-MAS) NMR has the advantage of measuring metabolites in intact tissues, thereby eliminating the tissue extraction process and preventing possible sample losses. Thus, this is an adequate tool for the investigation of endogenous metabolic changes [13]. Previous studies have reported the transcriptome [19,20] and proteome [21,22,23] of *V. harveyi*-infected shrimp. However, the few NMR metabolomics studies conducted on shrimp thus far have focused on *L. vannamei* exposed to sulphide toxicity [24], hypoxia [25], *Vibrio alginolyticus* [26], and white spot syndrome virus (WSSV) infection [27]. 

To the best of our knowledge, this is the first study to use HR-MAS NMR for the analysis of intact tissues of *V. harveyi*-infected *L. vannamei* at different timepoints. In shrimp, *V. harveyi* can usually be detected in the haemocytes [21], haemolymph [28], hepatopancreas [29], and gills [30]. Therefore, the gills, hepatopancreas, and haemolymph were chosen and analysed to observe the effect of *V. harveyi* infection on shrimp. The objective of this study was to establish a metabolic profile and investigate the endogenous metabolic changes in response to *V. harveyi* infection in shrimp in different tissues and at different timepoints.

## 2. Results

### 2.1. HR-MAS ^1^H-NMR Spectra of the Gills, Hepatopancreas, and Haemolymph of Whiteleg Shrimp

**Figure 1:**
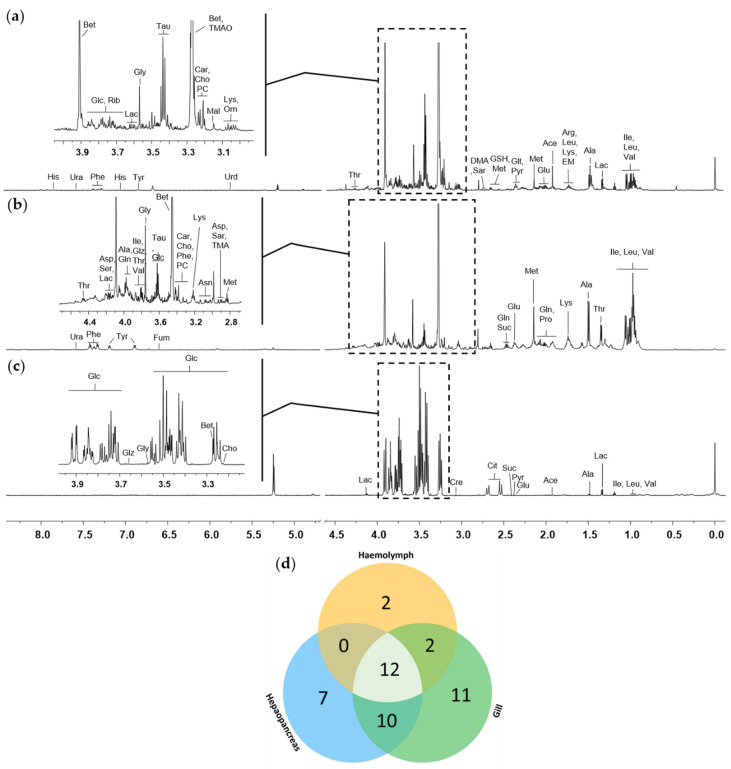
Representative ^1^H NMR spectra of intact gills (**a**), hepatopancreas (**b**), and haemolymph (**c**) tissues of whiteleg shrimp in the control group. The *y*-axis indicates peak intensity. Labelled peaks are as follows: Ace, Acetate; Ala, Alanine; Arg, Arginine; Asn, Asparagine; Asp, Aspartate; Bet, Betaine; Car, Carnitine; Cho, Choline; Cit, Citrate; Cre, Creatine; DMA, Dimethylamine; EM, Ethylmalonate; Fur, Fumarate; Glc, Glucose; Glu, Glutamate; Gln, Glutamine; Glt, Glutarate; GSH, Glutathione; Glz, Glycerol; Gly, Glycine; His, Histidine; Ile, Isoleucine; Lac, Lactate; Leu, Leucine; Lys, Lysine; Mal, Malonate; Met, Methionine; PC, O-Phosphocholine; Orn, Ornithine; Phe, Phenylalanine; Pro, Proline; Pyr, Pyruvate; Rib, Ribose; Sar, Sarcosine; Ser, Serine; Suc, Succinate; Tau, Taurine; Thr, Threonine; TMA, Trimethylamine; TMAO, Trimethylamine N-oxide; Tyr, Tyrosine; Ura, Uracil; Urd, Uridine; Val, Valine. (**d**) Venn diagram of the number of identified metabolites in the gills, hepatopancreas, and haemolymph.

The typical HR-MAS ^1^H-NMR spectra derived from the gills, hepatopancreas, and haemolymph of shrimp are shown in Figure 1. A total of 35, 29, and 16 metabolites were identified in the gill, hepatopancreas, and haemolymph tissue samples, respectively. The identified metabolites are broadly affiliated with carboxylic acids, amino acids, quaternary ammonium salts, amines, fatty acids, carbohydrates, dicarboxylic acids, tricarboxylic acids, alpha hydroxy acids, alpha-keto acids, organosulphonic acids, aminoxides, and pyrimidines (Table S1). The number of metabolites identified varied in a tissue-specific manner, as shown in Figure 1d. Twelve metabolites were common among the gill, hepatopancreas, and haemolymph samples. The gills shared 10 and 2 metabolites with the hepatopancreas and haemolymph, respectively, whereas there was no common metabolite between the hepatopancreas and haemolymph.

### 2.2. Multivariate Analysis of the Gills, Hepatopancreas, and Haemolymph of Whiteleg Shrimp

**Figure 2:**
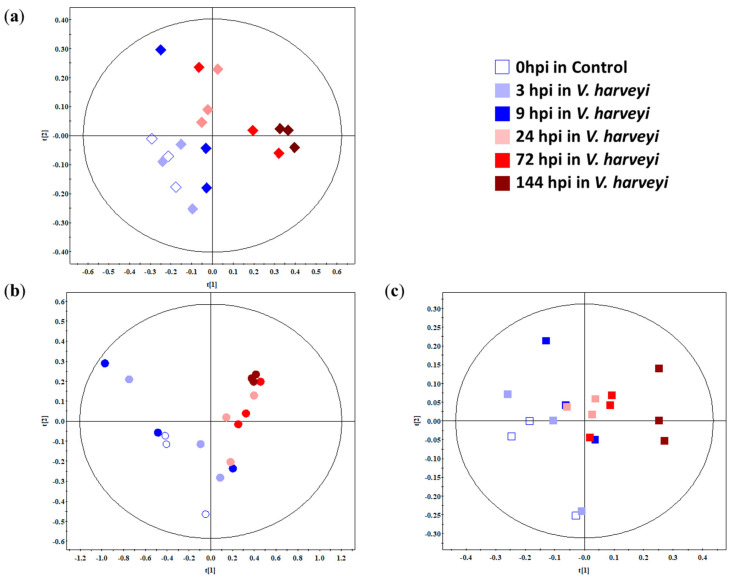
Partial Least Square (PLS) score plots based on the Nuclear Magnetic Resonance (NMR) spectra of the (**a**) gills (◇, diamond), (**b**) hepatopancreas (○, circle), and (**c**) haemolymph (□, square) from the whiteleg shrimp (*Litopenaeus vannamei*) infected with *Vibrio harveyi*. The shapes in diamond, circle and square represent samples in gills, hepatopancreas and haemolymph, respectively. The classification parameters for the PLS score plot were as follows: R^2^X = 0.987, R^2^Y = 1 and Q^2^ = 1, R^2^X = 0.986, R^2^Y = 1 and Q^2^ = 1 and R^2^X = 0.982, R^2^Y = 1 and Q^2^ = 1 for the gills, hepatopancreas and haemolymph, respectively. The colour represents hours post injection to *V. harveyi* in shrimp. □, 0 hpi in the control group; ■, 3 hpi; ■, 9 hpi; ■, 24 hpi; ■, 72 hpi; ■, 144 hpi in the *V. harveyi* infected group.

Partial Least Square (PLS) scores of the gills, hepatopancreas, and haemolymph samples were plotted on the NMR spectral data from the control and *V. harveyi*-infected group (Figure 2). Principle Component Analysis (PCA) scores were plotted to analyse the clustering of the samples in an unsupervised way and support the PLS score plot. They were plotted with six classes (according to time-point; 0, 3, 9, 24, 72, and 144 hpi). PCA and PLS score plots are represented in Figure S1 and Figure 2, respectively. The infection was divided into three phases on the gills and haemolymph: initial (3 and 9 hpi), mid (24 hpi), and late (72 and 144 hpi) with reliable Q^2^ value= 0.986 in the gills (Figure 2a), and initial (3 hpi), mid (9, 24, and 72 hpi), and late (144 hpi) stage in haemolymph (Q^2^ = 0.982, Figure 2c). A clear separation was observed between the early (3 and 9 hpi) and mid-to-late (24, 72, and 144 hpi) stages of *V. harveyi* infection in the hepatopancreas (Q^2^ = 0.986, Figure 2b) of whiteleg shrimp along [x = 0], longitudinal axis. Variable Importance in Projection (VIP) based on PLS was calculated to see the metabolites contributing for the separation among samples. A VIP score greater than 1.5 was selected and listed in Table S2. 

### 2.3. Relative Metabolite Concentrations in the Gills, Hepatopancreas, and Haemolymph of V. harveyi-Infected Whiteleg Shrimp

**Figure 3:**
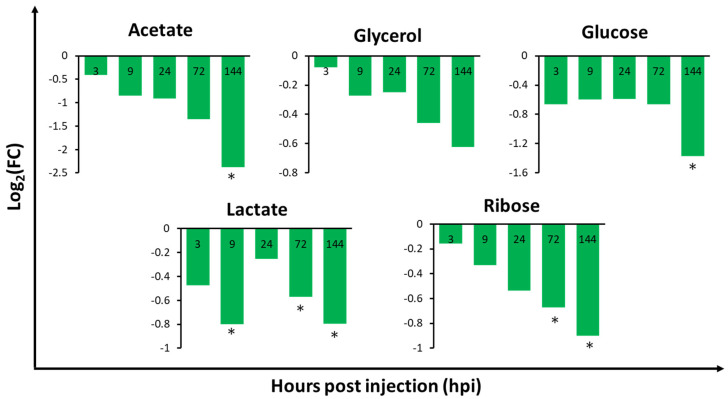
Fold change of relative metabolite concentrations in the gills of whiteleg shrimp (*Litopenaeus vannamei*) infected with *Vibrio harveyi* when compared to 0 hpi in the control group. Values with an asterisk (*) indicate significant differences when compared to 0 hpi in the control group (*p* < 0.05) (*n* = 3).

**Figure 4:**
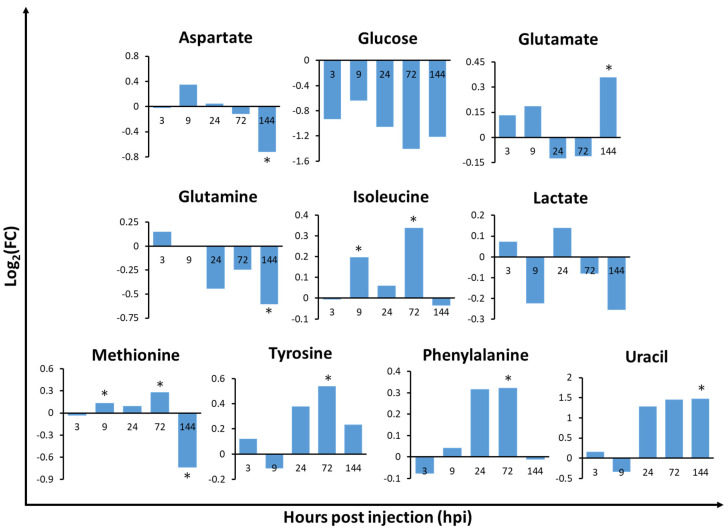
Fold change of relative metabolite concentrations in the hepatopancreas of whiteleg shrimp (*Litopenaeus vannamei*) infected with *Vibrio harveyi* when compared to 0 hpi in the control group. Values with an asterisk (*) indicate significant differences between the control and infected group (*p* < 0.05) (*n* = 3).

**Figure 5:**
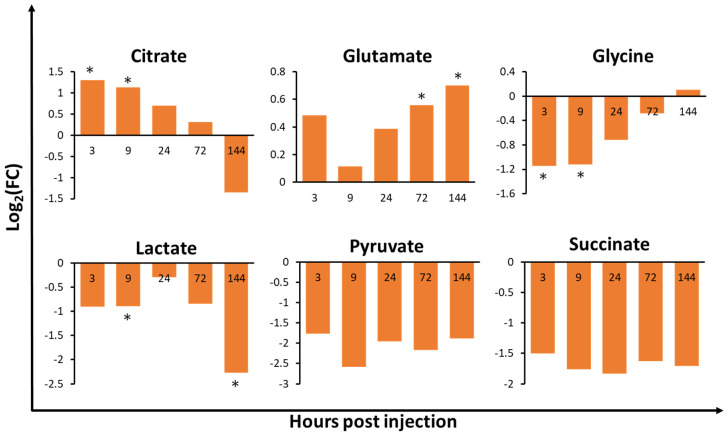
Fold change of relative metabolite concentrations in the haemolymph of whiteleg shrimp infected with *Vibrio harveyi* when compared to 0 hpi in the control group. Values with an asterisk (*) indicate significant differences between the control and infected group (*p* < 0.05) (*n* = 3).

The hepatopancreas of *V. harveyi-*infected shrimp showed the most significant metabolic changes at every time-point when compared to the other tissues analysed (Table S3), suggesting that the hepatopancreas is one of the most sensitive tissues to *V. harveyi* infection. In the haemolymph, 3 and 4 out of the 16 identified metabolites were significantly different at 3 and 9 hpi, respectively. This indicates that metabolic changes in the initial stage of infection occur more rapidly in the haemolymph than in other tissues. In the gills and hepatopancreas, metabolite concentrations exhibited significant changes as the infection progressed, especially at 72 (5/35 and 6/29 metabolites in the gills and hepatopancreas, respectively) and 144 hpi (6/35 and 12/29 metabolites in the gills and hepatopancreas, respectively). Notably, we observed dramatic changes in metabolite levels in the gills of infected shrimp at 144 hpi, such as a significant decrease in acetate and glucose levels by 2.38 log_2_FC and 1.37 log_2_FC, respectively (Table S3). In the hepatopancreas, we observed an opposite pattern of metabolic changes in most amino acids at 9 to 24 hpi when compared to 0 hpi. Uracil was significantly increased at 144 hpi, with 1.47 log_2_FC. In the haemolymph, citrate levels were found to be significantly increased (1.3 log_2_FC) at 3 hpi, while betaine levels were shown to be significantly decreased (1.5 log_2_FC) at 9 hpi. Lactate levels were also significantly decreased at 144 hpi, with a 2.3 log_2_FC. The lactate and ribose levels in the gills (Figure 3), isoleucine, and methionine levels in the hepatopancreas (Figure 4), and citrate, glutamate, glycine, and lactate levels in the haemolymph (Figure 5) exhibited significant changes in at least two timepoints. Moreover, the changes were shown to follow a specific pattern as the infection progressed.

### 2.4. Metabolic Pathway Analysis of V. harveyi-Infected Whiteleg Shrimp

To understand the affected metabolic pathways of *V. harveyi*-infected shrimp tissues, pathway analysis was performed. The changes in significant metabolic pathways based on the Kyoto Encyclopedia of Genes and Genomes (KEGG) database (*p* < 0.05 and impact > 0.1) differed in a tissue-specific and time-dependent manner (Table 1). The amino acid and carbohydrate metabolism was significant in the gills during the mid-late phase (72 and 144 hpi) of infection. In the hepatopancreas, the amino acid metabolism was mainly significant at 144 hpi. Interestingly, the pyrimidine metabolism in nucleotide metabolism was significant at 144 hpi of infection in the hepatopancreas. Uracil and glutamine were hit in this pathway, and, while glutamine decreased, uracil increased by approximately 1.5 log_2_FC from 24 hpi. In the haemolymph, the carbohydrate metabolism was the most significant, despite the amino acid metabolism. Moreover, the carbohydrate and amino acid metabolism was significant in the initial-to-mid phase of infection (3, 9 and 24 hpi). Hit metabolites (Table 1) involved in these metabolic pathways were related to carbohydrate and amino acid metabolism (Figure S2). Metabolites in Figure 3, Figure 4 and Figure 5 were selected based on hit metabolites in Table 1 and metabolites that show a specific pattern (e.g., continuous increase of decrease) as the infection progressed. 

#### 2.4.1. Metabolic Pathways Related to Carbohydrate Metabolism in the Tissues of Infected Whiteleg Shrimp

After *V. harveyi* infection, citrate and glycine levels were significantly altered during the early stage of infection (Figure 5) in the haemolymph. Notably, whereas the log_2_FC value of citrate decreased, that of glycine increased as infection progressed (Figure 5). Glutamate levels in the haemolymph also increased constantly after 9 hpi and were significantly different during the late stage of infection (Table 1 and Figure 5). The glycolysis/gluconeogenesis pathway was significant at 144 hpi only in the gills. In addition, the citrate cycle (tricarboxylic acid; TCA cycle) pathway was significant at all timepoints except 72 hpi in the haemolymph (Table 1 and Figure S2).

#### 2.4.2. Metabolic Pathways Related to Amino Acid Metabolism in the Tissues of Infected Whiteleg Shrimp

The metabolic pathways related to amino acid metabolism were confirmed mostly in the hepatopancreas and included phenylalanine, tyrosine, and tryptophan biosynthesis, phenylalanine metabolism, cysteine and methionine metabolism, and alanine, aspartate, and glutamate metabolism. (Table 1 and Figure S2). Fold changes in tyrosine and phenylalanine in the hepatopancreas showed similar trends, namely an increase in levels until 72 hpi, a significant difference at 72 hpi, and a decrease in levels at 144 hpi (Figure 4). Moreover, the tyrosine metabolism pathway exhibited significant changes in both the gills and hepatopancreas (Table 1 and Figure S2).

## 3. Discussion

This study aimed to investigate the physiological responses of whiteleg shrimp during *V. harveyi* infection using NMR-based metabolomics. Previous metabolomics studies of *L. vannamei* have mainly focused on the gills and hepatopancreas. Thus, the haemolymph spectra obtained in our study might provide fundamental data for further studies. In this study, betaine and taurine were found to be predominant in the gills and glycine, alanine, betaine, and the branched-chain amino acids, including leucine and valine, were found to be predominant in the hepatopancreas (Figure 1, Table S2). In the haemolymph, glucose was found to be the most predominant metabolite, accounting for approximately 93% of the total metabolites. While previous studies identified 16 [31], 22 [26], and 28 [27] hepatopancreas metabolites, we identified 29 metabolites from the intact hepatopancreas. The peak obtained for the hepatopancreas of *L. vannamei* by Liu et al. [27] was similar to that in our results, and glycine and betaine were also the most predominant, although the authors of that study used extracts of the hepatopancreas for the analysis. These results show that HR-MAS NMR spectroscopy permits the measurement of intact tissue without loss of metabolites. From the results of the PLS-DA score plot, concentration changes, and pathway analysis, we observed that the metabolic changes in *L. vannamei* after infection with *V. harveyi* were tissue-specific and time-dependent. 

In the gills, most of the energy-related metabolic pathways, such as pyruvate metabolism and glycolysis/gluconeogenesis, exhibited changes during the late phase of the infection (Table 1). Moreover, the amino acid metabolism was significantly altered at 72 hpi, and the carbohydrate metabolism was significantly altered between 72 and 144 hpi. This suggests that the significant changes observed in the gills, particularly during the late phase, might be related to the function of the gills. Aside from their major function as an osmotic and ionic regulator [32], the gills are also engaged in the identification and removal of foreign particles, especially bacteria, in the haemolymph [33]. As the infection progresses, the gills cannot continue to eliminate the *V. harveyi* pathogen, resulting in a high energy demand for survival. The levels of 8 out of the 18 amino acids identified in the gills were increased from 72 to 144 hpi when compared to 0 hpi. Increased amino acid levels in the gills were also observed in a study on the WSSV-infected gills of crayfish, which showed an enhanced energy metabolism [34]. In contrast, carbohydrate metabolism-related metabolites, such as acetate, lactate, and carbohydrates including glucose, glycerol, and ribose were decreased at all timepoints. Notably, we observed a dramatic decrease in acetate and glucose levels at 144 hpi. Yoganandhan and Thirupathi [35] have reported that the total carbohydrates and glucose levels were decreased in the tissues of WSSV-infected shrimp when compared to healthy shrimp. This suggests that amino acids may be accumulated for energy replenishment at a later time, while carbohydrate levels were decreased due to them being immediately used for energy. This is further supported by the fact that glucose, which is the major circulating carbohydrate in crustaceans [36], delivers immediate energy in the form of adenosine triphosphate by the process of glycolysis. Therefore, carbohydrates and amino acids are important for energy mobilisation in response to *V. harveyi* infection in shrimp. 

Compared to the other tissues, the hepatopancreas was found to have the most extensive metabolite changes and metabolic pathways, which suggested that the hepatopancreas is the hardest working tissue in infected *L. vannamei*. The amino acid metabolism was significantly changed in the hepatopancreas. Moreover, the turning point between early and late phase infection in the hepatopancreas seems to be between 9 and 24 hpi. Eighteen out of the 29 amino acid metabolites showed a different trend at 24 hpi when compared to other timepoints. Aspartate, glutamine, and glutamate levels tended to increase during the early phase and decrease during the late phase of infection. However, glutamate levels were shown to gradually increase from 24 hpi, being significantly increased at 144 hpi. Similar results were found by Su et al. [37], who showed that aspartate and glutamate levels were increased at 12 hpi (early infection) and decreased at 24 hpi (late infection) in WSSV-infected shrimp. Interestingly, isoleucine, methionine, tyrosine, and phenylalanine levels exhibited a significant increase at 72 hpi and then dramatically decreased at 144 hpi (Figure 4). Similar results were reported by Wu et al. [38], who found that amino acid levels were increased after WSSV-infection and decreased when the shrimp was close to death. However, more research is needed to further elucidate this mechanism. Glutamine and lactate levels were also found to be greatly decreased at 144 hpi in our study. To maintain the body energy consumption until the late phase of infection, most amino acids that were altered by metabolism (Table 1, Figure S2b) were metabolised into intermediates of the TCA cycle or to pyruvate, which in turn could be used as a precursor for gluconeogenesis. The decreasing trend in lactate levels from 72 to 144 hpi indicates that lactate contributes to the gluconeogenic supply of glucose for energy requirement in infected shrimp [27]. Wu et al. [38] reported that decreased glucose and lactate levels during the late phase of infection increases in gluconeogenesis in the hepatopancreas of shrimp. In addition, the nucleotide metabolism also exhibited unique changes in the hepatopancreas from 72 to 144 hpi. Apart from genetic information storage, some nucleotides, such as adenosine triphosphate and guanosine triphosphate, also act as energy carriers in the cell, providing energy for several cellular processes, including amino acid and protein synthesis [39]. The decrease in the levels of most amino acids at 144 hpi suggests that uracil is accumulated during the late phase of infection for amino acid synthesis. Thus, *V. harveyi* infection might alter the nucleotide metabolism to synthesise amino acids in order to compensate for the energy requirement during the late phase of the infection. In this study, when compared to the other tissues analysed, the hepatopancreas was found to exhibit the most changes as the infection progressed. The hepatopancreas of shrimp is reported to be the target organ of most bacterial pathogens [40]. Khimmakthong et al. [41] also showed that hepatopancreas is a target tissue in *Vibrio parahaemolyticus*-infected *L. vannamei*. However, more studies, such as histopathological and gene expression studies, are required. Regardless, our results suggest that the hepatopancreas can be a useful target tissue for further studies on *V. harveyi*-infected *L. vannamei* applying metabolomics approaches.

The haemolymph is important in shrimp with an open circulatory system. Since the *V. harveyi* injection in this study was performed intramuscularly, the pathogen penetrated the circulating haemolymph, which thereby exhibited changes faster than the other tissues analysed [42]. The carbohydrate metabolism in the haemolymph was mainly altered at 3, 9, and 144 hpi, and levels of related metabolites, such as lactate, pyruvate, and succinate, showed a constant decrease after infection. From 3 to 9 hpi, the glutathione and glycine, serine, and threonine metabolic pathways were affected (Table 1). Glycine was hit in all these metabolic pathways (Figure S2). Previous studies have reported that fluctuations in the level of glycine and glutamate in the haemolymph are involved in the general stress response in crustaceans [43]. Glutamate is considered as an amino-group donor for alanine, aspartate, and glycine synthesis [44]. The opposite trend of glycine and glutamate suggests that glutamate was accumulated for glycine synthesis (Figure 5). Thus, the increasing trend in both glycine and glutamate levels might be to satisfy the energy requirement in response to stress. One of the apparent representative characteristics of stress responses is the mobilisation of energy [45]. Aquatic crustaceans, such as shrimp, need to generate extra energy to cope with stress [44,46]. Our results showed that at 144 hpi, the TCA cycle in the haemolymph was uniquely altered (Table 1c, Figure S2a). Citrate is an intermediate metabolite of the TCA cycle. The main function of citrate inside the cell is an important regulator for the energy production since it induces pivotal enzymes found at the entrance or the exit of glycolysis, TCA cycle, and gluconeogenesis [47]. It is reported that supplementation of citrate is to promote the growth performance on shrimp [48]. Citrate was significantly increased during the early phase of infection (3 and 9 hpi) and then showed a decreasing trend from 3 to 144 hpi. The results were similar for pyruvate, which is the starting substrate for the TCA cycle. Downregulation of the TCA cycle suggests a disturbance in the synthesis of energy-related metabolites. The energy metabolism dysregulation caused significant alterations in *V. harveyi*-infected shrimp, with citrate and succinate levels decreasing by −1.3 log_2_FC and −1.7 log_2_FC, respectively. 

## 4. Materials and Methods 

### 4.1. Experimental Animals

Healthy whiteleg shrimp (body weight = 15 ± 1.0 g) were obtained from a commercial farm located in Chungcheongnam-do, South Korea and were acclimatised to a 110 L aerated seawater tank at 27–28 °C for 14 days. During the acclimatisation period, 10% of the water volume was changed daily. The shrimp were fed daily with a quantity of commercial feed equivalent to 3% of their body weight (kg). After acclimatisation, the shrimp were randomly divided into two groups (50 individuals/tank), namely the control and the *Vibrio harveyi* infection group. All experiments were performed in triplicate. All animal experimental procedures were carried out in accordance with the guidelines and regulations and with the ethical approval from the Ethics Committee of Pukyong National University (approval number: 2017–2010).

### 4.2. Bacterial Challenge

The *V. harveyi* used in this study was isolated from a diseased shrimp in an aquaculture farm located in Chungcheongnam-do in 2015. *V. harveyi* was grown in tryptic soy broth (Difco) for 24 h at 28 °C, then washed twice and diluted in sterile phosphate-buffered saline (PBS). For bacterial challenge, the shrimp in the infection group were injected with 100 µL of bacterial suspension (3 × 10^7^ CFU/mL) intramuscularly. The shrimp in the control group were injected with the same volume of PBS. The injected shrimp were returned to the 110 L aerated seawater tank at 27–28 °C and monitored daily for 7 days without feeding. A volume equivalent to 10% of the water volume in the tank was changed daily, and dead shrimp were removed from the tank as soon as they were found to maintain water quality. The cumulative mortality at day 7 post-injection was 9.3% and 3.3% in the *V. harveyi*-infected group and control group, respectively (Figure S3).

### 4.3. Sample Preparation

Three shrimp from each group were randomly sampled at 0, 3, 9, 24, 72, and 144 h post-injection (hpi), and haemolymph and tissue samples from the gills and hepatopancreas were taken aseptically. A volume of 400 µL of haemolymph was collected from the ventral sinus and transferred immediately to 1.5 mL micro-centrifuge tubes. The haemolymph, gill, and hepatopancreas samples were immediately frozen in liquid nitrogen and stored at −80 °C prior to NMR analysis.

### 4.4. ^1^H-NMR Measurements

The intact tissues of gill and hepatopancreas, and haemolymph were analysed using HR-MAS NMR. Twenty-five milligrams of each tissue (gill and hepatopancreas) was put into a nanotube (Agilent Sample Tube, 4 mm) containing 25 μL D_2_O supplemented with 2 mM sodium 3-trimethylsilyl-2,2,3,3-d_4_-propionate (TSP-d_4_). Forty-five microliters of haemolymph mixed with 5 μL of D_2_O containing 20 mM TSP-d_4_ was used for the ^1^H-NMR measurements. The ^1^H-NMR spectra were obtained using the Agilent 600 MHz NMR spectrometer (Agilent Technologies, Santa Clara, CA, USA) equipped with a 600 gHX NANO NMR probe with the spinning rate of 2050–2060 Hz at 298 K. The Carr–Purcell–Meiboom–Gill pulse sequence with presaturation of water resonance was used to remove the macromolecular compounds and water peak. The NMR spectra were acquired using 128 scans, an acquisition time of 3.0 s, total echo time 64 ms, and a relaxation delay of 3.0 s, with the total time being 13 min 9 s.

### 4.5. Spectral Pre-Processing

The obtained ^1^H-NMR spectra were processed using the Fourier transformation method, and the baselines were adjusted to minimise quantification errors by using the Chenomx NMR Suite 7.1 software (Chenomx Inc., Edmonton, AB, Canada). It is the most widely used in the study of metabolites that is an integrated tool for identifying and quantifying 1D NMR spectra. The reference compound was TSP-d_4_ and the concentration of metabolites were automatically calculated from TSP peak integral. Each peak of the ^1^H-NMR spectra was normalised to the total area. Fitting of signals, quantification, and assignment of each metabolite was processed using Chenomx NMR Suite 600 MHz library database as described by Mercier et al. [49]. The library comprises hundreds of fully searchable pH dependent compound models which enables the peak identification reliable [49]. For the ^1^H-NMR spectra of the gill samples, the region between 0.6 ppm and 7.95 ppm was binned, with a bin size of 0.001 ppm, and the water region between 4.5 and 4.8 ppm was removed from the analysis. For the ^1^H-NMR spectra of the hepatopancreas samples, the area between 0.6 ppm and 7.66 ppm was binned, with a bin size of 0.001 ppm, and the water region (4.6 ppm to 4.85 ppm) and the spinning side band (6.6 ppm to 6.68 ppm), which was created due to incomplete averaging were excluded. For the haemolymph samples, the ^1^H-NMR spectra were binned from 0.6 ppm to 6.55 ppm, with a bin size of 0.001 ppm. The water (4.5 ppm to 4.9 ppm) and spinning side band (1.1 ppm to 1.24 ppm, 1.7 ppm to 1.85 ppm, and 3.622 ppm to 3.672 ppm) regions were excluded. 

### 4.6. Multivariate Statistical Analysis

Using the SIMCA P+ 12.0 software (Umetrics, Umea, Sweden), we performed a PCA, PLS, and VIP score for the above-mentioned binned spectra by applying a Pareto scale. A variable with a VIP score greater than 1.5 was considered as an important metabolite. The results of PCA and PLS were visualised using a score plot to differentiate between the control and the *Vibrio harveyi*-infected groups. All metabolite concentrations are shown as mean ± standard deviation. A *p*-value of < 0.05 was considered statically significant when using two-tailed t-test for each time-point compared to the 0 hpi in the control group to examine the effects of bacterial infection on metabolites.

### 4.7. Pathway Analysis

To identify changes in metabolic pathways after bacterial infection, we performed pathway analysis using the MetaboAnalyst 4.0 software (http://www.metaboanalyst.ca) [50]. For pathway analysis, the data for relative concentrations of each annotated metabolite were processed via normalisation to the total area and Pareto scaling. The KEGG pathways that hit two or more metabolites in each pathway and had a pathway impact of >0.1 and a *p*-value of <0.05 were considered statistically significant.

## 5. Conclusions

This study successfully revealed time-sensitive metabolic changes at certain timepoints (3 to 144 hpi) in the gills, hepatopancreas, and haemolymph of *V. harveyi*-infected shrimp through NMR metabolomics. Overall, the changes in metabolites suggested that *V. harveyi* increased the energy demand in *L. vannamei* from early to late phase infection. Each tissue and haemolymph exhibited different metabolic changes depending on the progress of the infection. Thus, rather than focusing on a single tissue, future studies should consider conducting systemic profiling and analysis. We believe that this study will provide fundamental information about shrimp affected by infectious diseases and help monitor their health.

## Figures and Tables

**Table 1:** Significant Kyoto Encyclopedia of Genes and Genomes (KEGG) metabolic pathways (*p* < 0.05 and impact > 0.1) of the gills (**a**), hepatopancreas (**b**), and haemolymph (**c**) in *L. vannamei* infected with *Vibrio harveyi*.

(**a**)									
**Metabolism**	**Pathway**	**3 hpi**	**9 hpi**	**24 hpi**	**72 hpi**	**144 hpi**	**Hit**	**Metabolite(s)**	**Impact**
Carbohydrate metabolism	Pyruvate metabolism	-	-	-	0.041	0.012	3/22	Pyruvate, Lactate, Acetate	0.24
Carbohydrate metabolism	Glycolysis / Gluconeogenesis	-	-	-	-	0.020	3/26	Pyruvate, Lactate, Acetate	0.13
Amino acid metabolism	Tyrosine metabolism	-	-	-	0.045	-	2/33	Tyrosine, Pyruvate	0.13
(**b**)									
**Metabolism**	**Pathway**	**3 hpi**	**9 hpi**	**24 hpi**	**72 hpi**	**144 hpi**	**Hit**	**Metabolite(s)**	**Impact**
Amino acid metabolism	Tyrosine metabolism	0.009	-	0.004	-	0.0005	2/33	Tyrosine, Fumarate	0.19
Amino acid metabolism	Cysteine and methionine metabolism	-	0.045	-	-	0.0002	2/33	Serine, Methionine	0.14
Amino acid metabolism	Phenylalanine, tyrosine and tryptophan biosynthesis	-	-	0.012	-	0.005	2/4	Tyrosine, Phenylalanine	1.00
Amino acid metabolism	Phenylalanine metabolism	-	-	0.012	-	0.005	2/8	Tyrosine, Phenylalanine	0.38
Amino acid metabolism	Alanine, aspartate and glutamate metabolism	-	-	0.019	0.005	0.014	7/27	Aspartate, Asparagine, Alanine, Fumarate, Glutamine, Glutamate, Succinate	0.61
Nucleotide metabolism	Pyrimidine metabolism	-	-	-	-	0.003	2/41	Glutamine, Uracil	0.10
Metabolism of other amino acids	D-Glutamine and D-glutamate metabolism	-	-	-	-	0.034	2/6	Glutamate, Glutamine	1.00
Carbohydrate metabolism	Glyoxylate and dicarboxylate metabolism	-	-	-	-	0.049	4/32	Serine, Glycine, Glutamate, Glutamine	0.19
(**c**)									
**Metabolism**	**Pathway**	**3 hpi**	**9 hpi**	**24 hpi**	**72 hpi**	**144 hpi**	**Hit**	**Metabolite(s)**	**Impact**
Carbohydrate metabolism	Glyoxylate and dicarboxylate metabolism	0.033	0.028	0.021	-	0.024	4/24	Acetate, Citrate, Glycine, Glutamate	0.17
Carbohydrate metabolism	Citrate cycle (TCA cycle)	0.04	0.034	0.02	-	0.024	3/20	Citrate, Succinate, Pyruvate	0.17
Metabolism of other amino acids	Glutathione metabolism	0.041	-	-	-	-	2/26	Glutamate, Glycine	0.13
Amino acid metabolism	Glycine, serine and threonine metabolism	-	0.011	-	-	0.036	5/33	Betaine, Choline, Creatine, Glycine, Pyruvate	0.29
Carbohydrate metabolism	Pyruvate metabolism	-	-	-	-	0.013	3/22	Acetate, Lactate, Pyruvate	0.24

## Supplementary Materials

The following are available online at https://www.mdpi.com/2218-1989/10/6/265/s1, Figure S1: PCA score plots, Figure S2: Metabolic pathways in carbohydrate metabolism and amino acid metabolism categories in tissues of *V. harveyi* infected shrimp, Figure S3: Cumulative mortality, Table S1: Classification of assigned metabolites in gill, hepatopancreas and haemolymph of *Litopenaeus vannamei*. Table S2: VIP scores, Table S3: The relative concentration (%) of metabolites and Log_2_ FC (fold change) value in the gill, hepatopancreas, and haemolymph (c) of shrimp after *Vibrio harveyi* infection.
