# Supplementary material for: Vibrio harveyi Infection Significantly Alters Amino Acid and Carbohydrate Metabolism in Whiteleg Shrimp, Litopenaeus vannamei
Source: Metabolites. 2020 Jun 25;10(6):265. doi: 10.3390/metabo10060265 (PMC7344672; doi:10.3390/metabo10060265)
Supplement: Supplementary file 1 [file metabolites-10-00265-s001.zip › metabolites-823594-supplementary_v.1/Table S2.pdf]

Supplementary Table 2

Table S2. Variable Importance in Projection (VIP) scores calculated by Partial Least Square (PLS).

Gill		Hepatopancreas		Haemolymph	
**Metabolites (ppm)**	**VIP score**	**Metabolites (ppm)**	**VIP score**	**Metabolites (ppm)**	**VIP score**
Betaine (3.256)	18.94	Betaine (3.249)	11.73	Glucose (3.256)	8.15
Taurine (3.249)	7.34	Isoleucine (1.279)	5.84	Citrate (2.537)	7.15
Acetate (1.91)	6.40	Taurine (3.417)	5.67	Glycine (3.556)	4.49
Carnitine (3.269)	5.74	Lactate (1.312)	3.91	Lactate (1.315)	3.88
PC (3.21)	5.13	Leucine (0.945)	3.86	Acetate (1.91)	2.29
Glycine (3.554)	4.25	Methionine (2.12)	3.86	Succinate (2.395)	2.21
Glucose (3.394)	4.18	Phenylalanine (3.115)	3.16		
Lactate (1.315)	2.79	Carnitine (3.213)	3.14		
Glycerol (3.555)	2.58	Glutamate (2.339)	2.14		
Ribose (4.096)	2.26	Tyrosine (6.879)	1.86		

*PC: O-phosphocholine
