# Supplementary material for: Vibrio harveyi Infection Significantly Alters Amino Acid and Carbohydrate Metabolism in Whiteleg Shrimp, Litopenaeus vannamei
Source: Metabolites. 2020 Jun 25;10(6):265. doi: 10.3390/metabo10060265 (PMC7344672; doi:10.3390/metabo10060265)
Supplement: Supplementary file 1 [file metabolites-10-00265-s001.zip › metabolites-823594-supplementary_v.1/Table S1.pdf]

Supplementary Table 1

Table S1. Classification of assigned metabolites in gill, hepatopancreas and haemolymph of *Litopenaeus vannamei*.

Metabolite	HMDB ID	Class	Tissue
Alanine	HMDB0000161	Amino acids	Gill, hepatopancreas, haemolymph
Betaine	HMDB0000043	Amino acids	Gill, hepatopancreas, haemolymph
Choline	HMDB0000097	Quaternary ammonium salts	Gill, hepatopancreas, haemolymph
Glucose	HMDB0000122	Carbohydrates	Gill, hepatopancreas, haemolymph
Glutamate	HMDB0000148	Amino acids	Gill, hepatopancreas, haemolymph
Glycerol	HMDB0000131	Carbohydrates	Gill, hepatopancreas, haemolymph
Glycine	HMDB0000123	Amino acids	Gill, hepatopancreas, haemolymph
Isoleucine	HMDB0000172	Amino acids	Gill, hepatopancreas, haemolymph
Lactate	HMDB0000190	Alpha hydroxy acids	Gill, hepatopancreas, haemolymph
Leucine	HMDB0000687	Amino acids	Gill, hepatopancreas, haemolymph
Succinate	HMDB0000254	Dicarboxylic acids	Gill, hepatopancreas, haemolymph
Valine	HMDB0000883	Amino acids	Gill, hepatopancreas, haemolymph
Carnitine	HMDB0000062	Quaternary ammonium salts	Gill, hepatopancreas
Lysine	HMDB0000182	Amino acids	Gill, hepatopancreas
Methionine	HMDB0000696	Amino acids	Gill, hepatopancreas
O-Phosphocholine	HMDB0001565	Quaternary ammonium salts	Gill, hepatopancreas
Phenylalanine	HMDB0000159	Amino acids	Gill, hepatopancreas

Sarcosine	HMDB0000271	Amino acids	Gill, hepatopancreas
Taurine	HMDB0000251	Organosulfonic aicds	Gill, hepatopancreas
Threonine	HMDB0000167	Amino acids	Gill, hepatopancreas
Tyrosine	HMDB0000158	Amino acids	Gill, hepatopancreas
Uracil	HMDB0000300	Pyrimidines	Gill, hepatopancreas
Acetate	HMDB0000042	Carboxylic acids	Gill, haemolymph
Pyruvate	HMDB0000243	Alpha-keto acids	Gill, haemolymph
Arginine	HMDB0000517	Amino acids	Gill
Dimethylamine	HMDB0000087	Amines	Gill
Ethylmalonate	HMDB0000622	Fatty acids	Gill
Glutarate	HMDB0000661	Dicarboxylic acids	Gill
Glutathione	HMDB0000125	Amino acids	Gill
Histidine	HMDB0000177	Amino acids	Gill
Malonate	HMDB0000691	Dicarboxylic acids	Gill
Ornithine	HMDB0000214	Amino acids	Gill
Ribose	HMDB0000283	Carbohydrates	Gill
Trimethylamine N-oxide	HMDB0000925	Aminoxides	Gill
Uridine	HMDB0000296	-	Gill
Asparagine	HMDB0000168	Amino acids	Hepatopancreas
Aspartate	HMDB0000191	Amino acids	Hepatopancreas
Fumarate	HMDB0000134	Dicarboxylic acids	Hepatopancreas
Glutamine	HMDB0000641	Amino acids	Hepatopancreas
Proline	HMDB0000162	Amino acids	Hepatopancreas
Serine	HMDB0000187	Amino acids	Hepatopancreas
Trimethylamine	HMDB0000906	Amines	Hepatopancreas
Citrate	HMDB0000094	Tricarboxylic acids	Haemolymph
Creatine	HMDB0000064	Amino acids	Haemolymph
