# Supplementary material for: Vibrio harveyi Infection Significantly Alters Amino Acid and Carbohydrate Metabolism in Whiteleg Shrimp, Litopenaeus vannamei
Source: Metabolites. 2020 Jun 25;10(6):265. doi: 10.3390/metabo10060265 (PMC7344672; doi:10.3390/metabo10060265)
Supplement: Supplementary file 1 [file metabolites-10-00265-s001.zip › metabolites-823594-supplementary_v.1/Figure S1.pdf]

Supplementary Figure 1

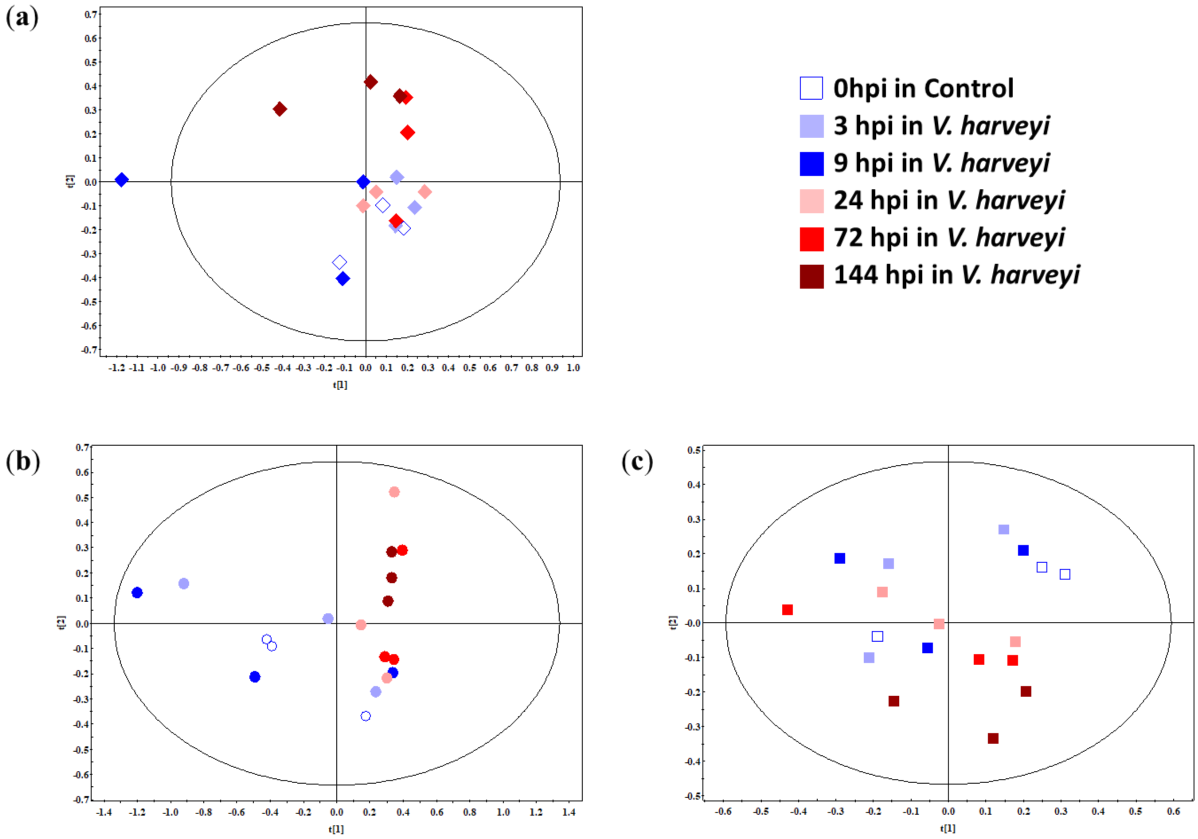

Figure S1. Principle Component Analysis (PCA) score plots based on the Nuclear Magnetic Resonance (NMR) spectra of the (a) gills (◇, diamond), (b) hepatopancreas (○, circle), and (c) haemolymph (□, square) from the whiteleg shrimp (*Litopenaeus vannamei*) infected with *Vibrio harveyi*. The classification parameters for the PCA score plot were as follows: R^2X = 0.711 and Q^2 = 0.335, R^2X = 0.926 and Q^2 = 0.627, and R^2X = 0.836 and Q^2 = 0.203 for the gills, hepatopancreas and haemolymph, respectively. □, 0 hpi in the control group; ■, 3 hpi; ■, 9 hpi; ■, 24 hpi; ■, 72 hpi; ■, 144 hpi in the *V. harveyi* infected group.
