# Supplementary material for: Vibrio harveyi Infection Significantly Alters Amino Acid and Carbohydrate Metabolism in Whiteleg Shrimp, Litopenaeus vannamei
Source: Metabolites. 2020 Jun 25;10(6):265. doi: 10.3390/metabo10060265 (PMC7344672; doi:10.3390/metabo10060265)
Supplement: Supplementary file 1 [file metabolites-10-00265-s001.zip › metabolites-823594-supplementary_v.1/Figure S2.pdf]

Supplementary Figure 2

(a)

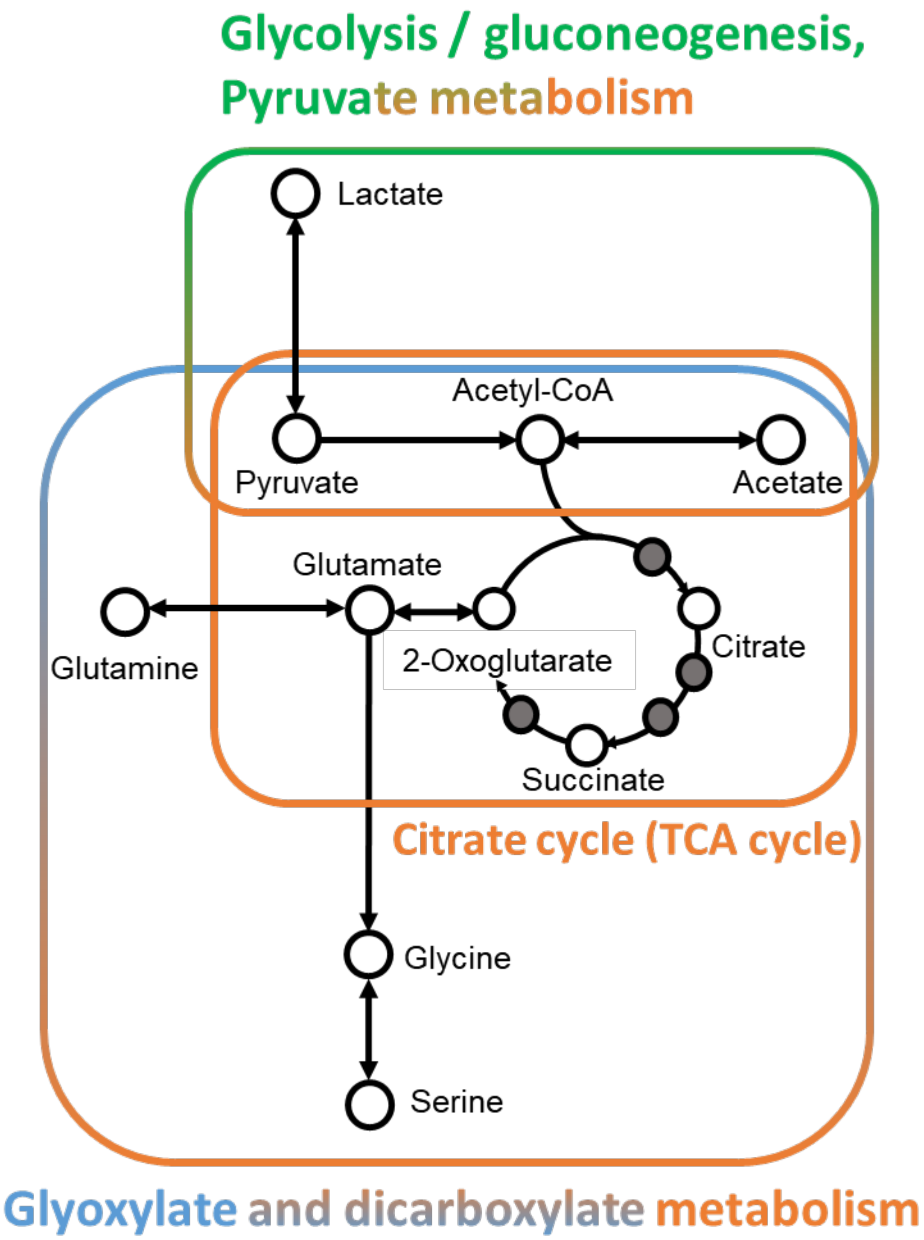

(b)

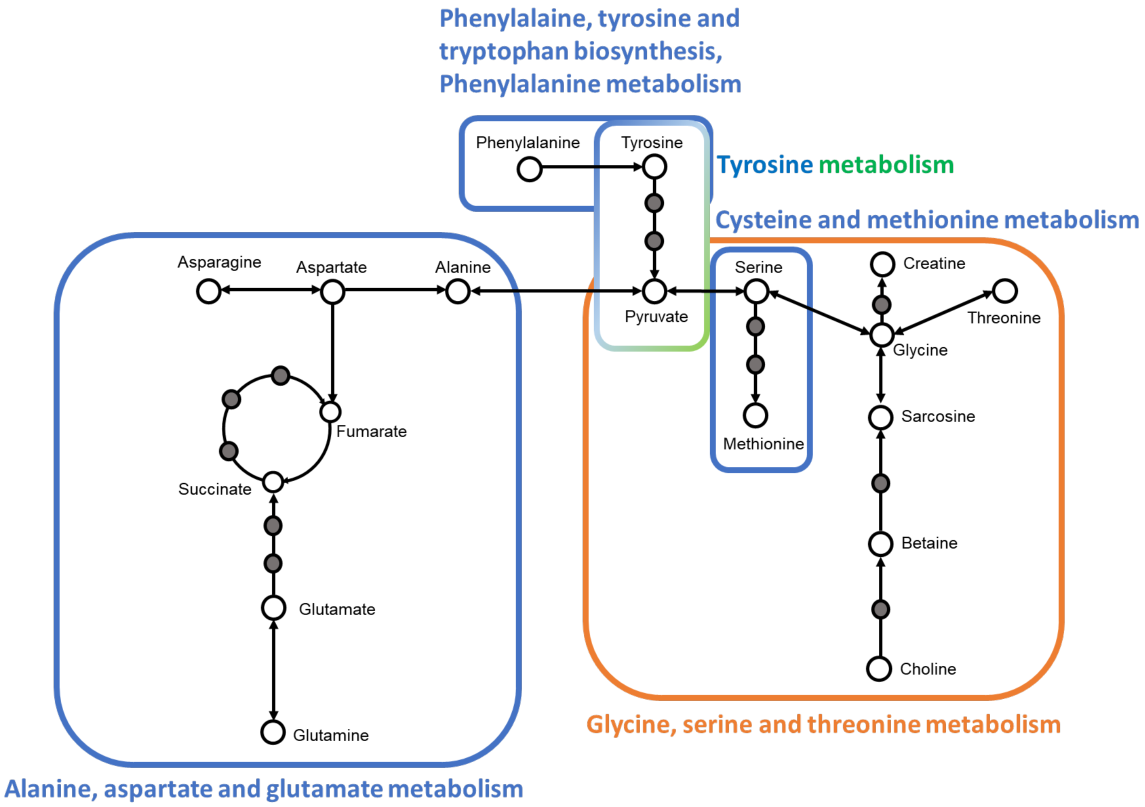

Figure S2. Metabolic pathways related to carbohydrate metabolism (a) and amino acid metabolism (b) in tissues of *V. harvey*i infected shrimp. The changes in each tissue was illustrated as color: gill, green; hepatopancreas, blue; hemolymph, orange. The mixed colour represents the pathway changed in common. The grey circle represents the intermediates between metabolites.
