# Supplementary material for: Vibrio harveyi Infection Significantly Alters Amino Acid and Carbohydrate Metabolism in Whiteleg Shrimp, Litopenaeus vannamei
Source: Metabolites. 2020 Jun 25;10(6):265. doi: 10.3390/metabo10060265 (PMC7344672; doi:10.3390/metabo10060265)
Supplement: Supplementary file 1 [file metabolites-10-00265-s001.zip › metabolites-823594-supplementary_v.1/Figure S3.pdf]

Supplementary Figure 3

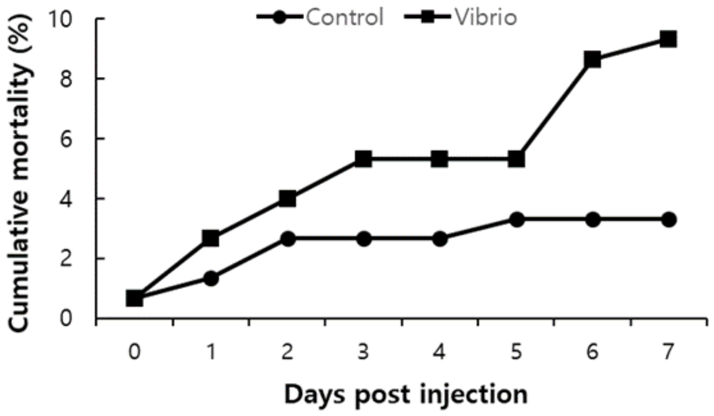

Figure S3. Cumulative mortality of control (Control) and *Vibrio harveyi* (Vibrio) infected groups. The final mortality reached at 3.3 and 9.3% at 7 days in control and *V. harveyi*-infected groups, respectively.
